# Editorial and Miscellaneous

**Published:** 1861

**Authors:** 


### EDITORIAL.

*Explanatory.*—Circumstances of a personal nature over which the acting editor had no control, delayed the issue of the June No. of the Journal so late in the month that it was concluded to issue the June and July Nos. together as a double number. Vexatious delays have again been our lot, but the double number is finally out, and we trust will be found valuable and interesting on perusal. For the future we remark that, having wholly revised our publication arrangements, there will be a prompt issue each month, We have assiduously endeavored to supply all missing Nos. to those applying, and steadfastly believe that by our perfected arrangements, there will hereafter be no omissions from the mailing list.

*Surgeons for the Army.*—In view of the vast number of the profession now overflowing with patriotic impulses, it is suggested that the various applicants for appointments to the surgical ranks of the army be mustered into distinct regiments, or at least companies like the sappers and miners.— Let them be armed with long catlins and pill boxes, and when in the actual face of the enemy, demand of them, with imprecations as terrible as whilom used in Flanders, that they shall swallow the contents of the latter—or be carved by the former. No opposing force could withstand them—the effect would be surer than bullets, more disastrous than bayonets.

*Apropos.*—Has there been any response to the fervent appeal of the Dix-ie nurse at Cairo, for a liberal supply of rags for the salivated soldiery in the military hospitals of that delectable seat of war? Our spirit groans and our heart is disquieted within us, when we think of our gallant soldiers who went out from us to defend the nation and in quest of glory, and have secured places in the hospitals and, in this June 1861--calomel sore mouths, necessitating an appeal to the city and country for store of rags to absorb the abominable flux thereof. In the name of all the gods of war, where are the results of our much lauded Examining Board, now gone into melancholy retiracy?

Will a calomel sore mouth entitle to a pension and bounty land? Will its bestowal entitle the attending surgeon to a medal—leather or otherwise? We pause for a reply.

---

*Measles.*—It is not a little remarkable that in the various camps, both north and south, the most prevalent disease by all odds has been measles. The very general employment of vaccination has thus far deprived variola of its traditional terrors. But for rubeola we have, at present, no reliable prophylactic. The only recourse, therefore, is to judicious regimen and treatment. Now, strange as it may appear, the treatment adopted is almost as diverse as the different camps where the disease prevails. In their commendable anxiety to distinguish themselves, it is to be hoped that our military faculty may not be tempted to employ the old, exploded heroic, perturbating methods. Perfect cleanliness, good ventilation, very little medication and a few days time, are all that is necessary for the vast majority of cases. Don't fill the patient's stomach with drugs, or his bowels with slops—give him fresh air and clean sheets. These are the essentials of good treatment.

---

*Dysentery.*—This very common disease of camps begins to show itself in the hospital wards. Very serious difficulty is apprehended by some of the fraternity, at home, because the non-intercourse and blockade orders have largely diminished the supply of Ol. Terebin thinæ. North Carolina, the principal source of supply of this potent engine of attack upon the bowels and divers and sundry of their ailments, has by the strong hand of military power been shut up from a large share of "the rest of mankind." Alas for the distressed intestines of the soldiery, unless the stigma of contraband is removed from this wondrous remedy. We are comforted in the midst of our griefs by the strong probability that our friend of the Nashville Journal, very much addicted to Turpentine in Typhoid fever, by virtue of the secession of Tennessee, need not be restricted in his use of this incomparable agent. We are waiting to see whether dysentery can be met and conquered without this very badly smelling and tasting elixir of life. Possibly it may be, and for ourselves, penitently and in all humility, we throw ourselves into the extended arms of the profession, and confess *ab imo pectore*, sinful soul that we are, that we believe ninety nine cases out of the hundred are better treated without than with the oozing abomination of the North Carolina pines. Free ventilation,clean sheets, a freely operating but mild laxative, once in twenty-four or at the most forty-eight hours,and then steady impression of general and local anodynes the balance of the time,being careful not to starve the patient if the disease persists beyond a few days—these measures we believe will more speedily and certainly carry the patient through, whether in camp, city or country, than all the calomel and turpentine secreted from the bowels of the earth or the woods of North Carolina.

Of the other fashionable method of treatment—salines, we have only to say, it will do if you urge not the salines to sensible effect upon the intestines too often.

---

*Dilatation of the Cervix uteri.*—Prof. Beverly Cole, of the Med. Dep. of the Univ. of the Pacific states that having failed in the use of the ordinary cones of compressed sponge he was led to prepare his own tents as follows:

"Select a fine piece of cup or surgeon's sponge, having melted a quantity of blanched beeswax in an ordinary vessel, the sponge is to be dipped into the liquid wax, and immediately placed between two smooth surfaces, (board or marble,) and a weight applied sufficient to compress the sponge and free from the surplus wax; in a few minutes it will be ready for use. By this process a flat cake of compressed sponge is obtained, from which pieces may be cut of such size and at such times as required. The piece to be introduced should be well oiled and carried to the point of strictures by means of a long and slender forcep."

---

*Pitting in Small Pox.*—Dr. Stokes concludes: 1st. That the chances of marking are much greater in the sthenic or inflammatory, than in the asthenic or typhoid confluent small pox. 2d. That considering the change in the character of disease observed during late years, we may explain the greater frequency of marking in former times. 3d. That in the typhoid forms of the disease the treatment of the surface by an artificial covering, such as gutta percha or collodion, will often prove satisfactory. 4th. That in the more active or non-typhoid form the use of constant poulticing, and of every other method which will lessen local inflammation, seems to be the best method of preventing disfigurement of the face.

---

*Question of Ethics.*—A correspondent wishes to know "whether it is good ethics for a physician claiming an honorable place in the regular profession to advertise with a "puff" in his county paper, that he is a regular physician, and gives special attention to diseases of the lungs, and diseases of women?" The reply is forced upon us that he violates the spirit and letter of the "code." Nevertheless the practice has become so common, even in large cities, and among the notorieties of the profession, that the question resolves itself practically into one of aesthetics rather than ethics. In our opinion, it is vastly more creditable to openly advertise and honestly pay for such a notice, than to toady and bribe the "locals" of the city and country press to lug one's name into the "dreadful accident" and "remarkable events" columns, as the manner of some is.

We never could see any particular superiority in dignity over *dead-heading* printers, editors, devils and reporters, in practice for the sake of being characterized in the newspaper columns, as the eminent, well-known, distinguished, skillful, experienced and learned Dr. So-and-so"—and in writing out one's own advertisement and paying for it like a man. As to the specialism involved, that is all *gammon*, of course, as the profession and intelligent part of the community already well know.

---

*Climatic Influence.*—The climate of California which seems remarkably favorable to recovery from the effects of severe wounds and surgical operations, according to the testimony of Dr. E. S. Cooper, appears to aggravate the effect of injuries of certain fibrous tissues. Sprains and bruises about the joints are exceedingly intractable, kindling up disease very apt to involve the subjacent bones. So also injuries of the periosteum. Difficulties of this species, which would be accounted trivial in the eastern states, there become of serious moment, and require extraordinary attention. Is this not attributable to the peculiar desiccant influence of the California atmosphere? In our own practice we find that persistent warm or even hot and moist applications are preferable to all others in relieving the effects of sprains and bruises in the region of joints or osseous surfaces.

---

*The Placenta and the Phenomena connected with the animal and organic nervous system.*

*The modus operandi of various kinds of baths, sea-bathing, heat and cold, physiologically explained.* Monographs by John O'Reilly, M. D. 1861.

The author of these monographs has been singularly industrious in prosecuting his researches into the phenomena of the nervous system. Without developing any essentially new ideas upon the subject, he has nevertheless succeeded in clearly stating propositions which are too often veiled in voluminous obscurity of language. It will be impossible for us to follow him in anything like an analysis of the pamphlets before us. In fact they scarcely claim to be more than analyses themselves. We need hardly do more than to direct the attention of that class of our readers who have devoted study to the subject, to these and others of Dr. O'Reilly's essays as worthy of their perusal, not as containing views worthy of their adoption indiscriminately, but as both suggestive of thought, and as evidence that thoughtful investigators are gradually freeing themselves from bold chemical mechanical, or worse than either, vague and empty *immaterial* notions. Dr. O'Reilly has made several steps upward from the cloudy speculations, and unsupported cob-webs of Prof. Paine, towards those clearly developed expositions as of the mechanism of nervous action which the latter gentleman characteristically misunderstood, and, misrepresenting, reached forth his skinny and ink stained digits to claim the offspring of his own brain.

There are, however, a few points in these pamphlets before us which we incidentally notice.

1. The author appears to recognize only stimulation and sedation of the nerves.

2. He speaks often of "contraction and relaxation of nerves."

3. That medicines act as antidotes.

4. That all nervous action is reducible to increase or diminution of motility in capillaries or nerves.

5. That poisons do not act by absorption but on the organic nerves.

In replication we aver:

1st. That the phenomena witnessed are wholly inexplicable unless something more than a quantitative change is admitted.

2d. That the phrase "contraction and relaxation of nerves" has no warrant either in observation or theory.

3d. That medical action is multitudinously various, not merely antidotal.

4th. That modification of motility either in capillaries, muscle or elsewhere, is but a single incident of nervous action, and that not an essential one.

5th. That the instances cited, by the author himself, most positively and conclusively prove that the majority of poisons act by local or general absorption, or both.

Our own views have been long before the profession. With regard to them, although our rightful claim to priority of elucidation was warmly contested, *the evidences are of record*, and we "bide the time." It is not our taste to haggle over priorities, especially with those who have but a glimmering of the matter. But we do rejoice to see the evidences of gradual approximation to the level of the true doctrine of nervous action, as first generalized and expounded by the present writer thirteen years ago, and "impressed" upon all his writings and lectures since. With Dr. O'Reilly's acute mind and industrious habits, both of study and writing, we shall expect him within a very brief period to shake off totally the trammels thrown around him by speculatists of the Paine stamp, and prove himself what he is capable of being, what he yet is not, an original and independent thinker and discoverer. Let him recognize *the molecular changes* which precede and follow the nervous phenomenon. Let him observe the uniformity of conduct of the fibre with whatever tissue its extremities may be in contact. Let him observe that the connection of nerve fibre is no more intimate with the, so-called, nerve vesicle than with the other changing cells or fluids with which its extremities are in relation. Let him observe that the character of the change produced by nerve action depends, not upon the nerve itself, but upon *the structure* which it influences. Let him recollect that to influence this nerve fibre with whatsoever "stimulus" it must be reached. If by medicine, by local absorption, or carried thence by the moving fluids effecting molecular and consequent nerve changes elsewhere—medicating or poisoning the blood as it occurs.

The operation of medicines through the nervous system and by absorption, are not incompatible as the vitalists vehemently assert, and as Dr. O'Reilly, in spite of his own senses to the contrary, still seems to believe—they are positively necessary to each, coincident and correspondent. The time has gone by when it must be believed either that the body is all fluids or all nerves. Solidism and humoralism in these latter days have laid down together, and the true medical philosopher, humble as a little child before the teachings of nature, leads them in perfect harmony.

---

*Constitution and By-Laws of the St. Paul Academy of Medicine and Surgery.*—This neatly printed pamphlet, received from our esteemed friend Dr. D. W. Hand, evinces a state of facts exceedingly creditable to the flourishing city of St. Paul. Although numbering but nine members, at the end of a year from its initiation its permanency and success is established beyond the remotest doubt. Notwithstanding the monetary depression, the Society has been put in possession of two spacious gas-lighted rooms, suitably furnished, a superior microscope, an analytical chemical apparatus, an electrical machine, and has laid the foundations of a Medical Library. The Treasurer reports cash receipts during the year $620.72; expended, $462.93; balance on hand, $367.07. They adopt the National Code, and invite the visits of all traveling physicians.

This is the kind of Medical Society which we have long dreamed of as desirable—not a mere debating club, or where doleful and doubtful essays are read to yawning auditors; where dubious practice is bolstered up by lumbering and questionable relation of experiences; but where the solid foundations of study, observation and comparison may be laid. We commend the example of St. Paul to each town where congregate any nine regular physicians who really love the profession and do not merely choose to bray over its reform, elevation and dignity in very asinine fashion. Look at it—*nine* professional gentlemen, loving and seeking to honor their profession, neither start a reform school or a debating club, but sensibly contribute nearly *six hundred and fifty dollars*, in a single year, to get the means of doing something for the profession, the best and speediest method whereof being to bring themselves up to the level of the highest, by making use of the means which the fruitful science of the time commends to their hands.

What might not the city of Chicago do with similar effort and liberality?

Chicago to-day is the hot-bed of quackery in all its repulsive forms. The profession is torn with petty cliques, and stunned by the clamor of noisy "reformers." The pitiful followers of that pitifullest of all impostures, Homœopathy, receive here a countenance and support from many, even in the so-called higher classes of society, which make the intelligent members of the profession blush for human nature, and hang their heads for very shame that they have to compete for practice on that low level. We call upon the profession to assert itself, not by empty resolutions and kill-joy societies, but by imitating the example worthily held up to them by the little city of St. Paul. Anybody can talk—Good Heavens, we have had talking and writing enough—now who will do something? Meanwhile, St. Paul, here is our ☞.

---

*Third Annual Report of the Chicago Charitable Eye and Ear Infirmary.*—This institution, organized and conducted under the auspices of a number of the leading, public spirited and philanthropic citizens of Chicago, still continues its humane and highly useful career. We learn: That during the year ending May 1, 1861, two hundred and eighty-eight patients have been under treatment; namely: two hundred and thirty-seven with diseases of the eye, and fifty-one with those of the ear; making an aggregate of five hundred and eighty that have been treated since the opening of the Infirmary, three years since.

The Trustees are highly gratified at the general success attending the treatment employed, and from the results feel warranted in urging attendance at the Infirmary by patients at as early period after attack of any of these various forms of disease as possible. Considering the deplorable effects of tampering with organs so important as the eye and ear, we look with great favor upon an institution, eminently calculated as this is to prevent unhappy patients from falling into the hands of the ignorant horde of professional oculists and aurists with which this, as well as all other large cities, abounds. We extract the following sensible and pertinent observations:

"The Surgeons would earnestly impress upon the minds of the trustees and of the public, that the great object of Charitable Eye Infirmaries is the *prevention* of calamities—the most terrible that can befall a human being. By furnishing the poor with gratuitous treatment for diseases of the eye, they remove the fear of expense, and thus encourage such patients to apply for medical aid in the earliest stages of disease, when most easily relieved. In this way they prevent not only blindness, but idleness and pauperism, with all their attending evils.

"Prevention of these evils is more politic, vastly more economical, and more in accordance with an enlightened humanity, than efforts to alleviate their sad and deplorable effects, after they once exist. Even on the ground of economy alone, is it not a matter worthy the careful consideration of all tax-paying citizens, that more than four thousand poor patients are annually treated at the New York Eye Infirmary, at an expense of only about *one dollar each*, while a class of less than one hundred pupils is maintained in the Blind Asylum of this State at an expense of not much less than *two hundred dollars each!*

Trustees—Walter L. Newberry, Flavel Moseley, William H. Brown, Samuel Stone, Charles V. Dyer, Dr. John Evans, Luther Haven, Cyrus Bentley, Ezra B. McCagg, John H. Kinzie, William Barry, Philo Carpenter.

Board of Surgeons—Consulting Surgeons, Prof. Daniel Brainard, M. D., Prof. Joseph W. Freer, M. D.; Attending Surgeons, Edward L. Holmes, M. D., Edwin Powell, M. D., Trustees Ex Officio.

The Dispensary of the Infirmary, in Ewing's Block, corner of North Clark and North Water streets, is open daily, from 11½ to 1 o'clock, for the graituitous treatment of the poor, afflicted with diseases of the Eye or Ear.

*The Right Man for the Right Place.*—The attention of the reader is especially solicited to the article which is reproduced on other pages from the *Am. Med. Times.* Every word of it is pregnant with truth, and as *pat* to this meridian as that of New York. With the exception of Prof. F. H. Hamilton, appointed to the Thirty-First N. Y. Regiment, we scarcely know of a surgeon of general eminence who is to be found in the military corps of the grand army of the United States. In the Southern army it is understood that this is quite different. Are not the surgeons of the Union as patriotic as those of the "Confederacy,"—or are the several State Boards of Medical Examiners, thus far selected, of such a character that self-respect will not permit either to go before them, or afterwards put themselves on the level of the "irregular practitioners, retired physicians, disabled political doctors, physicians unable to obtain a livelihood from sheer incapacity (who) have emerged from the Green Room, full fledged Army Surgeons?"

We might go a step farther than our *confrere* of the *Times*, and inquire: Are the Examining Board of the regular army up with the times, or do they survive in rank and position by virtue of the *prestige* acquired many years ago? The piping times of peace, during which these estimable gentlemen have been smoking their cigars and drinking their claret, have been prolific of medical advancement and discovery. We do know some that have kept pace therewith and some—quite otherwise.

It is true that the examinations for the regular service are conducted with very considerable vigor, but if *wrong answers are required to the right questions*, the applicant had better not pass, either for his own good or that of the unhappy soldiers who are committed to his charge. We admire a steady conservatism which prevents the incursions of wild theory and causeless innovation in practice, but we do not admire, or love or in any wise revere that stony inertia which resists all progress, and disowns all advance, which ignores all discovery and permits no place to new methods. Let the profession be represented, at all events in the volunteer service, by men alive to the influences of the times, whilst thoroughly grounded in the wisdom of true science. Let the eminent men, not merely notorieties, be so indicated by professional opinion, that they can, without dishonor or discredit, accept positions in the army service without being obliged to seek back-stairs political influences, and running a tilt with all sort of competitors, like applicants for Washington clerkships and four-corner post offices. As it is now we see little hope of escape from the dilemma, unless the general government takes the matter wholly out of the hands of the petty politicians, who, for the most part, at the present time occupy the several Gubernatorial chairs in the loyal States.

---

*Puerperal Convulsions.*—A medical friend in the city has detailed to us, within a few days, a recent case of severe puerperal convulsions wherein venesection to the extent of thirty ounces, in an apparently favorable case, produced no mitigation, but rather an aggravation of the symptoms, but which was speedily relieved by Chloroform. The day for reliance upon bleeding in this distressing malady has gone by. Chloroform is now the central remedy.

---

*A Treatise on the Practice of Medicine.* By Edwin R. Maxson, M. D., formerly Lecturer on the Institutes and Practice of Medicine in the Geneva Medical College. Philadelphia: Lindsay & Blakiston; 1661. 8 vo., pp. 705. From the Publishers.

This is another of the strictly American works, the number and character of which, we are happy to see, are gradually becoming more respectable.

In the very modest preface, the author informs us that this volume is essentially a resume of his course of lectures at the Geneva Medical College, written during scanty leisure hours snatched from the duties of an active professional life. It is therefore a transcript from the record of experience of an intelligent, well educated and successful practitioner, giving upon every page distinct evidence that while the author is familiar with the literature of the profession, he is at the same time imbued with strong practicality of views, which prevents him from being a mere compiler or rehearser of old time or new time dogmas. We do not hesitate to prophesy that "Maxson's Practice" will become a popular volume with practitioners, although not characterized by any strikingly original views in a strictly scientific sense.

In the discussion of each subject the author glances at the anatomy and physiology of the part treated of, being anxious "to have the mind of the reader fixed on the diseased part and its conditions." He observes: "I have attempted to draw up the work without even the shadow of empiricism, by taking the human system in health as the standard; and then noting the deviations from that standard, constituting the various morbid conditions or diseases. By taking this course I have been enabled to arrive at clear indications of treatment, from direct pathological conditions, for every prescription which I have made. This course by no means precludes the benefit of experience in the use of remedies; as remedies which are indicated from pathological conditions are always those which experience finds the most successful. I have preferred, then, to arrive at them in this way, rather than empirically, as it tends to lead the mind of the student and practitioner of medicine to prescribe for *conditions*, without reference to names."

This is most irrefragable common sense, and precisely wherein most writers and teachers of practice fail. It is true, most will admit the correctness of the abstract proposition, but unfortunately each one is wedded to some theoretic figment, or fastened astride of some hobby-notion, which vitiates too too many of his conclusions. Dr. Maxson has pretty generally steered clear of this difficulty, and even where we fancy that we can see pretty strong indications of his adhesion to views we cannot ourselves receive, his keen practical sense has triumphed over his abstract opinion. It has been his ambition to furnish the profession with a useful book, indicating the rational progress of medicine, and not merely to attract attention and admiration by making it the vehicle of startling novelties and questionable methods. We venture to pronounce it, in the main, a success, and highly creditable to him both as an author and practitioner. It has been very little if at all heralded by anticipatory puffs or extravagance of laudation, but it will take rank at once among the *timely* books, and its author merit and receive the thanks of the working part of the profession.

The volume is printed in the usual excellent style of its distinguished publishers, but we regret to notice a very considerable number of typographical errors, which however a subsequent edition will of course correct. None of them, so far as we have noticed, are other than mere artistic blunders, the sense being uninjured. Written as the work is in the free almost conversational, style of the lecture room, there is at times a little looseness of expression, and occasional repetition, which we commend to the author for subsequent revision—we only mention them because we expect to see hypercritical reviews, with which unfortunately the medical world abounds, seize upon them triumphantly in order to make a glowing display of their own wisdom and littleness.—We do not like to favor snarling dogs with even a bone.

The work is divided into fifteen chapters, from the titles of which it will be seen that a general survey of the subject is given, viz:

Chap. I—Disease. Ch. II—Irritation, congestion, Inflammation. Ch. III—Fever. Ch. IV—General Fevers. Ch. V—Exanthematous Fevers. Ch. VI—General Inflammatory diseases. Ch. VII—Diseases of the Nervous System. Ch. VIII—Dis. of the digestive system. Ch. IX—Dis. of the respiratory system. Ch. X—Dis. of the circulatory system.—Ch. XI—Dis. of the Eye. Ch. XII—Dis. of the Ear. Ch. XIII—Dis. of the Skin. Ch. XIV—Dis. of the Urinary Organs. Ch. XV—Dis. of the Genital organs.

By the praiseworthy exclusion of either original or quoted theorizing from his pages, the author has found abundant space to notice this very extensive series of subjects quite satisfactorily, and to elucidate principles and methods of treatment, more nearly brought up to the level of the times than in any other book on the subject we now call to mind. In some particulars, however, we shall take occasion to dissent from his practice somewhat. Thus in the treatment of intermittent and remittent, we might remark that the doses of quinine are not by any means sufficient to cope with those diseases as they occur in the west and south—although they may answer well enough in the author's particular locality. We must not omit to state that the new remedies, and new forms of administration of old remedies, are fully brought forward into common use. We heartily commend this book to all of our readers.

---

*Bed Sores.*—Dr. Loomis Bauer has found the following ointment very serviceable in relieving bed sores:

℞ Glycerine optimæ, . . . . . . . . . ʒ v

Coque et adde, . . . . . . . . . . . . ʒ j

Amyli, . . . . . . . . . . . . . . . ʒ ss

Ext. Belladonnæ, . . . . . . : . . . gr. iij.

M. ft. ungt.

---

*En Rapport.*—A cotemporary Medical journal has a *spiritual communication* from a supposed defunct anatomist, late of this city, and more recently from Pike's Peak. It is supposed that in his gold digging in the latter region, he prosecuted his investigations to such an unusual depth, that instead of returning, he kept on and has enjoyed a resurrection among the antipodes. As usual in this kind of communications, the information imparted is of such questionable sort, that we are led to exclaim over the departed, as did the poetical philosopher over the still-born child:

> "When I think how quickly he was done for,

> I wonder what he was begun for!"

*Summer Course of Instruction in Rush Medical College.*—The Summer Course in Rush Medical College was conducted by the gentlemen having it in charge, without interruption, throughout the period for which it had been announced in the annual circular. It was in every way successful, and the students in attendance express themselves in high terms of commendation of the teachings of the several instructors. We cannot forbear saying thus much, for we have noticed that the organ of a little clique which lives only upon its hatred and misrepresentation of this old established institution, for a purpose easily to be surmised, gravely announces the *discontinuance* of the summer course in Rush Medical College. Such pitiful exhibitions of spleen are scarcely worthy of *some* of the gentlemen connected with even that little coterieof disappointed individuals. One of these persons, claiming a certain amount of moral and even religious character, positively informed a patient, a few days since, that he had never heard of Dr. ——, with whom he had been a colleague in medical teaching for the greater portion of the last ten years! A reduction to such extremities argues a great want of faith in the individual's own hoarse eloquence about the "elevation of the profession." We commend him to his "class-leader."

---

*Coffee as a Remedy for Pertussis.*—The Boston Medical Journal revives the practice of M. Jules Guyot, of administering strong coffee for whooping cough. In one case a little girl six years of age was immediately relieved by doses of a tablespoonful and a half of strong coffee sweetened, but with milk, three times daily. "She had not a single whoop, after she began to take it." Other cases of marked relief are reported. Good, pure and strong coffee is to be used, the limit of the dose being the unpleasantly stimulating effect. A child of eighteen months will take half a cup of pretty strong coffee without any noticeable injurious effect. A little more experience is needed, and meanwhile we say,—"Important if true."

*Quinine as a Prophylactic.*—The Cleveland *Med. Gazette* for July urges the use of quinine as a prophylactic to intermittent fever. It observes: "We believe that all troops, stationed or marching, through malarious districts should be compelled to take from four to six grains of quinine daily.—Particularly should this be enforced at such places as Cairo, and by all sentries exposed at night, in localities where malarial diseases are prevalent. Every physican who has practiced in the great malarial districts of the west, we presume, is acquainted with this fact."

We must be allowed to observe that our own experience, based upon some fifteen years practice in a highly malarious western district, (not in Chicago where agues are as "scarce as hens' teeth,") has convinced us that this proposition is too sweeping. Where soldiers or civilians are exposed to malarious influences but for a few days at a time the practice is perhaps well enough, but when they are to remain any considerable length of time we should be despoiled by this practice of a valuable remedy. The system rapidly becomes accustomed to quinine, as to other cerebro-spinants, and inordinate doses are readily borne and necessary after tolerance is once established by use. The great objection to quinine is not its producing congestions or organic changes, but the evanescence of its influence. Scarcely any remedy is sooner climniated, and the simple "anti-spasmodics" assafœtida and valerian, do not sooner lose their power over the nervous system by repetition. Our own practice has invariably been to use quinine as an anti-periodic freely and in full doses, but not at all as a continuous remedy. The ordinary vegetable bitters and mineral acids are, in our experience, preferaable where a continuous impression is sought. The decoction or Tinct. of Cinchona with the addition of Aromat Sulph. Acid answers a good purpose, but we especially prefer the Dilute Nitric Acid in moderate doses. The important point is to keep the health of the soldier above the level of the disease, and this object is to be secured by hygienic rather than medicinal measures. But if any of the various forms of intermittent do occur, then arrest them at once—*at once*, with the quinine. Every hour the disease continues enhances the liability to a subsequent attack. Quinine will about infallibly do this, unless its efficiency has been previously exhausted by frequent use. We have scarcely met a case where by proper combination we have not been able to give this capital remedy as an anti-periodic. According to the circumstances of the particular case, any peculiar unpleasant effect it produces upon individuals may be counteracted, without impairing its febrifuge powers, by combination with the preparations of opium or other narcotics, capsicum, bisumth, hydrocyanic acid, antimonials, veratrum viride, alcohol, etc. None of these will prevent its anti-periodic impression. The great trouble in its use arises from the utter tolerance established by its continuous administration. Our opinion is summed up in the proposition that Quinine is inestimable and invaluable as an anti-periodic, and *ne plus ultra*.

---

*Compliment to the Medical Profession of the United States.*—The chairman of the great Sanitary Commission, advisory to the Medical Bureau of the Grand Army, is a Unitarian preacher, distinguished especially for his advocacy of theatres and dances as not necessarily mortal sins. It is understood that Phineas T. Barnum, Esq., and Mons. Blondin are to be honorary members, in consideration of the eminence they have respectively attained in their respectable avocations.—We respectfully beg leave to nominate the steamboat man, Geo. Law, Esq., and the renowned Christopher Carson, whilom guide to the Maj. Gen. John C. Fremont over the Rocky Mountains. Meanwhile we have not a word to say about the Rev. Dr. President of the Sanitary Commission, any more than about the Right Rev. Bishop of Louisiana, who has doffed the robes of the spiritual church militant for the togs and small sword of the carnal general of a militia division. Our only fear just now is that we may be forced from the medical editorial tripod to fill some vacant pulpit.

*New York Dental Journal.*—This very readable and valuable Journal is well worthy the support of our friends immediately occupied in that branch of remedial art. May we call the attention of its editors to the fact that in the July No. they have ascribed the article "Removal of Parotid Tumor" to R. L. *Mead*, M. D., etc., instead of R. L. *Rea*, M. D.; and that both that and the following article on Puerperal Convulsions should have been credited to the Chicago Medical Journal? We dislike particularly to have other parties held responsible for our utterances. The omission in this case is clearly through inadvertance, and hence we can allude to it with greater freedom than to many similar ones we could mention, where our ideas and expressions have been bodily appropriated.

---

*Iron in Cutaneous Diseases.*—Prof. Cooper recommends strongly the use of the Ferro Cyanuret of Iron in doses of from two to four grains, gradually increasing, three times a day to children of a year and upwards suffering from *tinea capitis*. The remedy should be continued if necessary for several months. Locally he applies every third or fourth day an ointment consisting of equal parts of camphor and tar ointment with the addition of an eighth part of chloroform.

In the varieties of Psoriasis seen in California he commends an aqueous solution of the Pot. Tart. Ferri: two ounces to three of water—to be well shaken and applied every day to patches. The same solution is found useful as a local application to phagaedenic ulcers.

---

*Eczema.*—Local applications of caustic potash in various degrees of solution according to the apparent depth of the affection are again rising in favor, scabs and scales if any, should first be removed by use of warm oil and spiritus saponatus.—Subsequent irritation to be relieved by warm or cold water dressings as may be indicated.

*Ephelis.*—This annoying affection so common during pregnancy, sometimes remaining in a chronic state long after, rarely requires constitutional treatment. Daily ablution of the part with a mild solution of carbonate of potash, and after thoroughly drying it, applying with a sponge a lotion composed of equal parts of lemon juice and proof spirit, will generally remove the discoloration in a few days. The lotion should be continued however for a considerable period to secure a complete cure. Should this fail, a week solution, say a grain to the ounce of water, of Hydrarg. Bi. Chlorid will probably succeed.

It may be mentioned that among the manifold uses of Chlorate of Potash, not an inconsiderable one is its efficiency in several varieties of cutaneous disease—this among others.—Acne is particularly amenable to it. A solution of thirty grs. to the ounce of water is a suitable strength for a lotion for eudermic application.

---

*Prurigo Pudendi.*—Local applications are of little avail, although in some cases temporarily palliative. The symptoms being evidently of a reflex character, probably dependent upon irritation of the os and cervix uteri, as might be easily made manifest we have for many years been in the habit of directing local applications to the os, and upper portion of the vagina, of Belladonna or other narcotic, either in the form of ointment, applied directly upon the part, first dried by a sponge; or what is more feasible in practice in aqueous solution by vaginal injection. Nearly the same effect can be produced by exhibition per rectum. A much smaller quantity, however is requisite in the latter case.

If the ointment is preferred, it should be applied very carefully over the os in the proportion of one part to five or ten of the cerate. The injection may be used in the strength of from five to twenty grains to the amount of menstrum employing two or three ounces at a time. The recumbent position should be continued for one or two hours. Should symptoms of constitutional affection be manifested, the parts should be freely washed so as to remove any portion of the medicine remaining. There is to be but little trouble to be apprehended from this cause, as much the larger proportion of the injection fails to be retained.

When the prurigo has been present for some considerable length of time the local change in nutrition in the labia is prone to exhibit itself in papules, aphthae and sometimes even very considerable thickening and induration. When these occur, the local treatment becomes useful as subsidiary, but cannot be relied upon for cure which requires removal of the cause. Several cases have fallen under our observation where caustic applications had been freely applied to the os without benefit, and indeed with some aggravation of the symptoms, but which were speedily relieved by the direct application of the Belladonna ointment. In our experience the carbonic acid gas has not proved of much service for this purpose. Chloroform proves too irritating, either in the form of vapor or as commonly diluted with Olive oil or Glycerine. Occasional advantage has been derived from the addition of from forty drops to a drachm to the ounce of Glycerine, or simple cerate, rubbed up with a few grains of morphine. In all cases of this troublesome difficulty too much stress can scarcely be laid upon the importance of careful regulation of alvine evacuations. Indeed very many cases require no other treatment. For this purpose laxatives of the mildest character are indicated.

---

*Inflammation of the Mamma during Lactation.*—Among the most common and troublesome of the dificulties of early lactation is inflammation of portions of the mammary gland. It seems upon observation of the methods of treatment in vogue that some practitioners overlook what is frequently the essential canse of the disease, to wit, a disordered condition of the uterus. An almost endless variety of local applications to the gland itsef are proposed, many of them well adapted to relieve the local difficulty, but in very many cases all these prove of little avail. Recourse must be had to measures which will correct the morbid state of the uterus or its appendages. In every case presented, this requires especial notice. The synergies of the uterus and mammae are perhaps more remarkable than any others in the system. The mere presence of the lochia, and absence of uterine pain or tenderness, will not alone disprove the presence of grave uterine irritation which is expended in reflex symptoms manifested in one or both of the breasts. In our experience, attention to the condition of the uterine organs furnishes the best prophylactic against mammary abscess. Refrigerant and sedative applications to the breast, keeping it lightly covered or wholly exposed, thoroughly drawn, and then relieving uterine disorder by such remedial agencies as may be indicated, have succeeded in preventing the formation of abscesses which seemed irrepressibly imminent. Whereas, in similar cases, reliance upon local treatment, of the most varied character, has wholly failed to prevent thei rformation. It is deemed unnecessary to do more than to call the attention of intelligent practitioners to this idea, in order to materially modify the practice too often depended upon.

---

*Abortive Treatment of Typhoid Fever.*—Prof. Austin Flint, in a recent clinical lecture, whilst admitting that his own ideas are not settled upon the point, nevertheless thinks that the results justify further trials of the remedies to this end. He appears to incline to the opinion that opium is the more important agent. Dr. O. C. Gibbs, in commenting upon this practice, suggests four-grain doses of opium with ten or twelve of quinine. He says: "The remedial powers of quinine, we are confident, are not sufficiently appreciated by the profession. This combination may not be adapted to all cases, but having given it in puerperal fever, pneumonia, and a variety of other febrile diseases, with a success that is satisfactory to us, we should not fear to give it a trial in nearly every case of typhoid fever. Such cases here have been remarkably few, but to such as have fallen under our charge in the last two years, we have given the remedies, in smaller doses it is true, but oftener repeated, and never to our regret."

In those cases where remittent fever assumes the continued or typhoid form, we have been in the habit of using this combination with great confidence and advantage, but in ordinary typhoid, whilst the opium has proved itslf in full doses an admirable agent, we have found little benefit from quinine, except, perhaps in the later stages of the disease. We have no confidence in any remedy yet presented to avert the disease, although we see no reason why something may not yet be discovered which will do the work. But judicious use of the means we already have will, in nine cases out of ten, conduct the patients to convalescence in from twelve to sixteen days. We have elsewhere enlarged upon this point.

---

*We live in Deeds not Words.*—A blatant medical reformer in this city distinctly and knowingly violated the "code" Sunday, July 21st, and Monday, July 22d inst, and this is with him an everyday occurrence, as many gentlemen of the profession in this city will testify whenever required. We give due notice to this gentleman, and any other especial advocates of the "elevation of the profession" that we shall make an *expose* of these unprofessional acts, unless a speedy stop is put to them. We suggest that it is better to elevate than to be elevated. Meanwhile "the rod is in pickle."

---

*Medical Communications and Proceedings of the Connecticut State Med. Society. Sixty-ninth Annual Convention, Hartford*, 1861.—This is a very interesting pamphlet, being the second number of the volume, the whole being now published so as to be preserved in a permanent form. President Woodward's Annual Address is upon the somewhat general subject—Life. Amid a variety of ingenious speculations of a highly interesting character,are interwoven many valuable suggestions of a practical nature, and doctrinal ideas worthy of reflection.

Dr. J. B. Lewis furnishes a well written article on hereditary predisposition ; and Dr. L. S. Wilcox a Sanitary Report for the county of Hartford, 1860. Dr. Wilcox from his statistics finds that while the average proportion of mortality of females to males under five years of age is as 100 to 110 8-100 that from this age to forty-five the proportionate mortality of females increases. The indications from these and other statistics is that taken together in all ages, nature very fairly maintains an equilibrium.

Several prominent medical men, now deceased, in the State are suitably memorized. The most generally eminent of those noticed is Prof. Wm. Tully. Prof. Tully was unquestionably one of the most generally learned men of our profession this country has known. His services to medicine more particularly consist in the result of his large attention to many indigenous remedies—we may mention Conium, Sanguinaria, Cimicifuga, Veratrum, Asclepias, etc., etc. With vast attainments, the mere stealings from which have made many little men notorieties, he had very little practical tact, and within our own memory failed as a successful general practitioner of the healing art. He was emphatically a man of books—a walking cyclopædia. Intensely wedded to his peculiar ideas, no unfortunate practical result whatever could shake his unwavering confidence in his preconceived theories. With little in his private deportment to attract friendship, he commanded respect by the unsurpassed extent and variety of his acquirements. His own life, like his semi-finished work on materia medica forcibly illustrates the *embarrass du richesse.* He never organized his knowledge into a faculty, or could wield it as a power. Thus his works, like his life, are almost invaluable to the medical scholar, but of limited worth to the plain practitioner.

We can put our hands upon at least half a score of gentlemen who have become the next thing to famous, by simple *absorption* from the writings of Tully.

Judging by its fruits the Connecticut State Med. Society is a prosperous and highly useful institution.

---

*Oil of Turpentine as an Anaesthetic.*—Among the manifold uses to which this article has been put, Mr. John Wilmshurst in a communication to the London Lancet, March 1861, introduces anæesthesia. He avers he has tried it, administered sprinkled upon a handkerchief, by inhalation, in several cases of severe neuralgia and minor surgical operations, and that it seems to allay pain, nervous irritation and spasm without deranging the action of the heart, and produces a calm anæsthetic sleep. *Credat Judœs Appella.*

---

*Teeth Drawing without Pain.*—Rub the gums with the following solution by means of a bit of lint or cotton steeped in it: ℞ Chloroform ℥ iss., Tinct. Aconite, Spts. Vini aa ℥ j Morph. Sulph. gr. viij M. For which the American Druggists' Circular is responsible.

---

*Epilepsy.*—Prof. McGugin, of the Iowa University, strongly recommends the Hydrocyanate of Iron as an empirical remedy, and says that several cases under his treatment had no doubt been cured by it. He first suggested its use for this purpose.

---

*Quinia.*—The Society of Pharmacy of Paris, have offered a prize of six thousand francs for the discovery of a substitute for quinine, or for a method of artificially forming it.

---

*The Year Book.*—Owing to the stringency of the times, and consequent limited number of subscriptions, Dr. O. C. Gibbs has postponed the issue of his contemplated "Year Book of American contributions to Medical science and literature.

*Medical Colleges.*—Apprehensions of unprecedented mortality exist among the colleges. The young gentlemen are either off to the wars, or are brief in finances from similar military causes. The peculiar position of Chicago is such, however, that we feel warranted in stating to that considerable portion of our readers who have an interest in the matter, that the prospects of Rush Med. College were never more flattering than at present. The Secretary informs us that the number of letters of enquiry received thus far, materially surpasses those at the same period last year. Notwithstanding this, however, we shall not be surprised to hear that the total number of medical students in attendance upon medical colleges throughout the country, will scarcely be more than half or two thirds the usual proportion. We hear that one of the prominent eastern schools which has usually had a class of not less than five hundred, thinks that it will do well if it secures one hundred and fifty the ensuing winter. It is to be hoped that in the competition thus likely to arise, there will be no further resort to the contemptible methods employed by sundry parties a year since in attempting to build up their own institution at the expense of an old established school? *Verb sap.*

---

*Army Surgeons at Manassas.*—It rejoiced the editorial heart to hear that Prof. A. B. Palmer, of the Mich. Univ., whilom of this city, by assiduous use of his legs, escaped being "forwarded to Richmond" subsequent to the recent disaster. What would the Am. Med. Association have done for a parliamentarian? Who would have aided * * * in poising the lever of "elevation" *under* "the profession?" We shudder at the remote suggestion.

---

*Unblushing Effrontery.*—A person, whom we shall not further characterize, publicly advertises in a prominent daily paper of this city, "A Retreat for Females,"—the character of which may readily be inferred from the following extract:

"Also those unfortunate cases of females who by 'deformed pelvis,' 'constitutional disability,' OR OTHERWISE, cannot with safety become mothers, will find the "Retreat" a safe place for their relief."

Comment is unnecessary, but meanwhile where are the Grand Jury?

---

*An Adroit Dodge.*—A contemporary medical publication carries upon its title page the name of a local *attache* of one of the city papers. It thus secures a temporary reprieve from summary dissolution, long threatened, and meanwhile the "local" aforesaid manufactures almost diurnal notices of the weakly "Reform School," to the especial interests of which our contemporary is devoted. A leading proprietor of the city paper involved stated the other day in our hearing, that the multitudinous notices referred to were admitted on the benevolent principle of helping the weakest party—"the under dog in the fight."

---

*Opposition Lines.*—Newspaper puffing, audacious mendacity; the assistance of graduated milkmen, whose untimely advent to the medical profession, the abandoned herds and, withal, mankind deplore; the assiduity of hired runners and *claquers;* the unscrupulous laying on of hands upon, as yet, unconsecrated theological students; the inveigling of incautious strangers to places they did not dream of seeking, may be material elements of professional "reform and elevation," but we have been accustomed to give them *other* names.

---

*Internal Application of Ice in Uterine Hæmorrhage.*—Our friend, Dr. Teal, of Albion, Ind, in a private letter, gives us the following note:

"A medical friend related to me the following case: "I was once put to my trumps while trying to repress uterine flooding in a case under treatment. I went out and cut a piece of ice to the size of a medium nutmeg. This I introduced, holding it between my fingers and letting it slip from them into the cavity and against the wall of the womb. The flooding instantly ceased."

We have no doubt that this mode of applying ice in controlling uterine hæmorrhage might be made available more frequently than it is. It is a favorite method with several practitioners of our acquaintance even prior to the use of the usual battery of appliances. It requires, however, care and judgment in its use, or subsequent inflammatory action may be lighted up. Ordinarily, we have found it free from any unpleasant results.

---

*Medical Journals and the War.*—The faces of our Southern exchanges are missed from the editorial sanctum, and we are free to say that no other contraband of war is more regretted in absence. We had little thought so soon to miss their genial pages, and their always instructive contents. Fare you well, dear Bowling, prince of jokers—Campbell the scholarly; Dowler, the indefatigably industrious; Byrd & Hauser, with the portrait of GEORGE MCLEILAN upon the title page of your capital monthly—farewell till we meet again, when the fife has done squeaking and the drums are done with their racket, when red-legged gentlemen no longer either fight or steal chickens, when doctors no longer rush before Examining Boards and thence to the tented field—when the preacher resigns the presidency of the Sanitary Commission, and the newspapers chronicle the exploits of the myriad M. D.'s, home from the wars. Until then—Farewell! We keep our *files* for you, not to spike your guns or ours, but to transmit by regular course of the UNITED STATES mail.

